# Disrupted establishment of anaerobe and facultative anaerobe balance in preterm infants with extrauterine growth restriction

**DOI:** 10.3389/fped.2022.935458

**Published:** 2022-09-06

**Authors:** Yi-E Huang, Xintian Shen, Dingding Yin, Shanwei Lan, Yongxue Lu, Ping Zhou, Liya Ma, Yinlan Zhang, Yuhui Sheng, Youjun Zhang, Mengna Li, Fei Hu, Jiaqi Chen, Pan Li, Emad M. El-Omar, Huimin Zheng

**Affiliations:** ^1^Department of Hospital Infection Control, Shenzhen Baoan Women's and Children's Hospital, Jinan University, Shenzhen, China; ^2^Department of Pharmacy, Shenzhen Baoan Women's and Children's Hospital, Jinan University, Shenzhen, China; ^3^Department of Neonatology, Shenzhen Baoan Women's and Children's Hospital, Jinan University, Shenzhen, China; ^4^Department of Obstetrics and Gynecology, The First People's Hospital of Foshan, Foshan, China; ^5^The Second Clinical Medical College, Southern Medical University, Guangzhou, China; ^6^Department of Neonatology, The First People's Hospital of Foshan, Foshan, China; ^7^UNSW Microbiome Research Centre, St George and Sutherland Clinical Campuses, UNSW Sydney, Sydney, NSW, Australia

**Keywords:** extrauterine growth restriction, preterm infants, obligate anaerobes, facultative anaerobes, longitudinal study, gut microbiota

## Abstract

**Background:**

Extrauterine growth restriction (EUGR) in preterm birth infants could have long-term adverse impacts on health. Less is known about the gut microbiota regarding its establishment in early life and its role in long-term growth in preterm birth infants.

**Methods:**

A prospective, longitudinal observational study was conducted with 67 preterm infants in a level III neonatal intensive care unit. Clinical information was obtained from medical records, and fecal samples were collected weekly during hospitalization and processed for 16S rRNA gene sequencing.

**Results:**

The bacterial profiles from the weekly sampling of preterm infants demonstrated that the early-life gut microbiota was clustered into the following four stages in chronological order: stage 1: 0–4 days, stage 2: 1–2 weeks, stage 3: 3–7 weeks, and stage 4: 8–10 weeks. The development of gut microbiota showed latency at stage 4 in EUGR infants compared with that in non-EUGR infants, which resulted from their consistently high level of facultative anaerobes, including Enterobacteriaceae and *Staphylococcus*, and lack of obligate anaerobes, including *Clostridium* and *Veillonella*. In the 2-year follow-up, infants with a high level of obligate anaerobes-to-facultative anaerobes ratio at stage 4 had a lower risk of long-term growth restriction at the margin of statistical significance.

**Conclusion:**

The results of this study indicate that the development of gut microbiota in the early life of EUGR infants is delayed compared with that of non-EUGR infants. The obligate-to-facultative anaerobes ratio could be an indicator of the maturity of gut microbiota development and associated with the risk of long-term growth restriction in preterm infants.

## Introduction

Preterm birth is one of the most common adverse pregnancy outcomes and a leading cause of neonatal mortality (1). Extrauterine growth restriction (EUGR), which is usually defined as discharge weight below the 10th percentile in postnatal neonates, remains a high morbidity in the neonatal intensive care unit (2) and correlates with disorders in multiple systems, including reduced glomerular filtration, affecting protein absorption of infants (3–5). Although parenteral and enteral nutrient supply could provide support for improving growth (6), EUGR preterm infants, particularly low-birth-weight infants, remain smaller than their term peers (7), which indicates that the provision of nutritional support is not sufficient to meet the needs of EUGR infants. The answer might hide in the gastrointestinal tract, where food is digested and commensal gut florae interact and grow together with the host.

Gut microbiota plays an important role in nutrient utilization and metabolism; therefore, the establishment of a healthy intestinal microbiota is beneficial in terms of the long-term wellbeing of these infants (8, 9). It has been reported that the establishment of gut microbiota in preterm birth infants was stunted, which was characterized by the reduced level of strict anaerobes. Commensal health-promoting bacteria, such as *Bifidobacterium* and *Lactobacillus*, remained in a relatively low abundance. However, with the development of the intestine, the *Clostridiales* were gradually elevated, meaning that obligate anaerobic bacteria increased (10). The dynamic of early life gut microbiota echoed the oxygen concentration and maturity of the gut. Obligate anaerobes and facultative anaerobes were observed in the early intestinal tract, and their content varies with oxygen concentration, but the relationship between the maturation of gut microbiota and the obligate-to-facultative anaerobes ratio is still unclear (11). Moreover, despite limited studies reporting gut microbiota shifted in EUGR infants, whether the establishment of gut microbiota is related to infantile growth and maturity remained unclear (12). In this study, we aimed to study the difference establishing pattern of gut microbiota between EUGR and non-EUGR preterm infants and explore the association between bacterial characteristics and the risk of short-term and long-term growth restrictions.

## Materials and methods

### Study participants

We recruited 67 preterm infants from the level III neonatal intensive care unit (NICU) of Shenzhen BaoAn Maternal and Child Care Hospital from August 2018 to July 2019. Infants born under 37 gestational weeks and with parental written informed consent were prospectively included, and those with congenital malformations, necrotizing enterocolitis, or who withdrew from the study during hospitalization were excluded. The weight of infants was measured daily during hospitalization and at 24 months old after discharge by follow-up visits. EUGR was diagnosed when discharge weight ≤10th percentile derived from Fenton's postnatal growth charts (2). Long-term growth restriction was defined as the weight at 24 months ≤10th percentile derived from the WHO Child Growth Standards (weight-for-age chart for boys and girls, respectively, 2006). Other clinical information obtained from medical records is described in the Methods in the Supplementary materials.

### Sample collection, DNA extraction, and sequencing

We collected meconium and stool samples (0–1 day) from preterm infants' soiled diapers. After the meconium collection, another stool sample would be collected in the middle of the first postnatal week (day 3 or 4) and then collected weekly (week 1–10) during hospitalization. DNA was extracted using the Minka Gene Stool DNA Isolation Kit (Magigene, Guangzhou), and the V4 region of 16S ribosomal RNA (rRNA) gene was amplified as previously described (13) and added negative samples in the steps. No statistical significance was detected after 30 cycles. The library preparation and sequencing were conducted using Illumina TruSeq Preparation Kit and MiSeq sequencer, respectively. The raw sequencing data are deposited in the EBI repository (https://www.ebi.ac.uk/) with accession number PRJEB45532.

### Statistical analysis

The 16S rRNA sequencing data were subjected to quality control, denoise (using dada2 with default parameters), taxonomic annotation (using RDP against GreenGenes Database v13_8), followed by the construction of an amplicon sequence variant (ASV) table, and α- and β-diversity calculation using QIIME2 according to its online tutorial (14). We totally obtained 11,533 ASVs, and 4,371 ASVs were discarded after standardizing to 6,440 reads for each sample, which is adequate according to rarefaction curves (Supplementary Figure S1). An independent validation cohort conducted by Duke University was downloaded from NCBI under BioProject PRJNA544545 (15) and processed with the same pipeline. The data of each sample are independent, and the sampling analysis is not repeated. Permutational multivariate analysis of variance (PERMANOVA) was employed to determine the explanation of clinical characteristics in bacterial sample dissimilarity (16). To identify the enriched bacteria between groups, we performed a linear discriminant analysis effect size (17) (LEfSe) between the EUGR and non-EUGR groups. We used multivariate association with linear models (MaAsLin2) to determine EUGR-associated genera in meconium after adjusting for delivery mode (18). Wilcoxon rank-sum tests were conducted to identify differences between the EUGR and non-EUGR groups. We used R package *pheatmap* to demonstrate the change of bacterial abundance and cluster of EUGR infant age. The cluster method of heatmap was calculated using complete-linkage clustering (setting “method='complete”') in R package *hclust*. BugBase was used to annotate potential oxygen phenotypes (19). Then, we used Spearman's correlation analysis to explore the correlations between infant chronological age and the abundance of each bacteria oxygen phenotypes. Logistic regression analysis was performed to access the odds ratio for long-term growth restriction according to the obligate-to-facultative anaerobes ratio at stage 4. More methodology details are described in the Methods in the Supplement materials.

## Results

### Study population

We enrolled 67 preterm infants. By comparing the discharge weight of infants with Fenton's postnatal growth charts, we divided participants into EUGR and non-EUGR groups (47 and 20 participants, respectively). Of note, 20 (29.9%) infants were born by vaginal delivery; 44 (65.7%) infants' weights were <1,500 g at birth; eight infants were diagnosed as small for gestational age; and all developed into EUGR. Early-onset infection occurred in 11 infants from the non-EUGR group (55.0%) and 30 infants from the EUGR group (63.8%). Detailed statistics along with gender, family, and feeding characteristics are reported in Supplementary Table 1. Among participants, weight information of 60 individuals was collected by visiting them for follow-up at 24 months old after discharge, and seven individuals (five from the EUGR group and two from the non-EUGR group) were lost to follow-up.

### Dynamics of the gut microbiota in EUGR preterm infants during hospitalization

The rarefaction curves illustrated the adequacy of sample size for 16S rRNA sequencing (Supplementary Figures S1A–C). We observed differences in beta diversity of microbiota but no significant difference in alpha diversity between EUGR and non-EUGR groups in the infants' meconium (0–1 day; Supplementary Figures S2A–E). Firmicutes, Proteobacteria, Actinobacteria, and Bacteroidetes were the majority phyla of meconium in the two groups (Supplementary Figures S2F, G). Proteobacteria (Enterobacteriaceae and Moraxellaceae) were significantly higher in the EUGR group than in the non-EUGR group and remained significant after adjusting for delivery mode (Supplementary Figure S2H, Supplementary Table S2).

**Figure 1:**
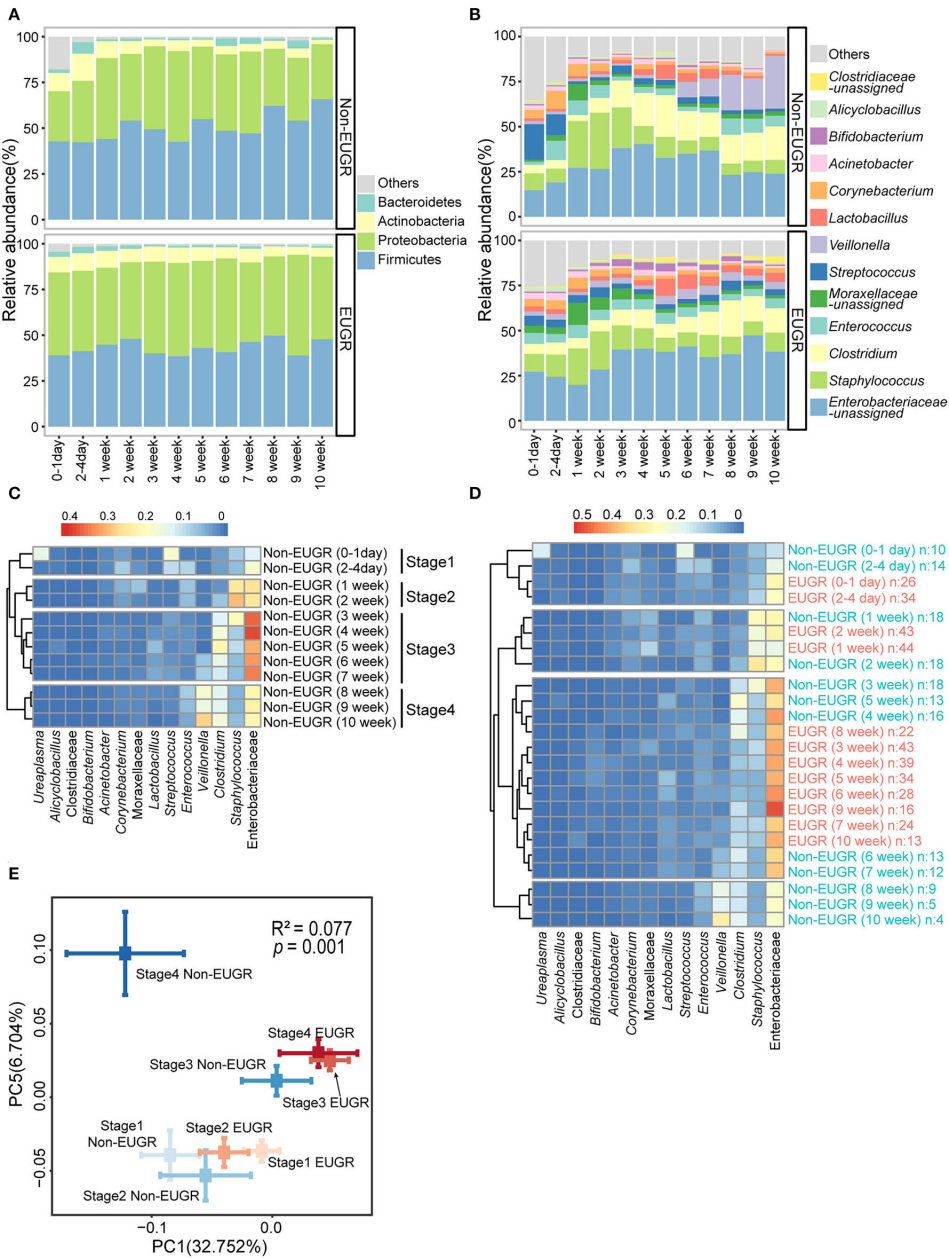
The gut microbiota patterns of preterm birth infants with and without EUGR. **(A,B)** Average relative abundances of predominant taxa at the phylum and genus levels in the EUGR and non-EUGR groups. **(C)** Heatmap shows the predominant bacteria in the non-EUGR group clustered by time into four stages. **(D)** Heatmap shows the predominant bacteria in the EUGR group clustered together with the non-EUGR group. **(E)** Principal coordinate analysis based on unweighted UniFrac distances showing the mean values and standard errors of the PC axes of different groups (mean ± SE). Differences in beta diversity among groups were tested using permutational multivariate analysis of variance.

From the longitudinal samples during hospitalization, we observed dynamic changes in phyla, mainly, the gradual enrichment of Firmicutes and depletion of Proteobacteria, in the non-EUGR group, while the bacterial composition remained relatively stable across time in the EUGR group (Figure 1A). The same trend was observed at the genus level. The relative abundance of *Enterobacteriaceae-unassigned* remained >25% from birth to discharge in the EUGR group (Figure 1B).

To study the dynamic patterns of bacteria, we first clustered the abundant bacteria from non-EUGR infants for each time point. As expected, four time-dependent stages were formed by the hierarchical clustering method (Figure 1C). In Figure 1C, we observed that the gut microbiota development of non-EUGR infants showed a chronological pattern. Four chronological stages were defined according to the clusters (stage 1: 0–4 days; stage 2: 1–2 weeks; stage 3: 3–7 weeks; and stage 4: 8–10 weeks). When we used the samples from the EUGR group to perform the clustering analysis with non-EUGR samples, we observed a delayed development in the EUGR group. The results showed that EUGR bacterial samples from 0 day to 2 weeks were clustered into stages 1 and 2, indicating a similarity in bacterial development in the early life. Interestingly, the rest of the EUGR time points from 3 to 10 weeks were all clustered into stage 3, while none were in stage 4 (Figure 1D). As shown in the heatmap, stage 3 was characterized by a high level of *Enterobacteriaceae-unassigned*, which was considered a sign of immaturity in the early-life gut microbiota (20). These indicated that the gut microbiota of EUGR preterm infants might suffer delayed progression in stage 4. To evaluate this tendency at the sequence level, we performed a principal coordinate analysis based on weighted UniFrac distance with EUGR and non-EUGR groups. Samples from the EUGR group were also assigned to these four chronological stages according to the sampling time. In the non-EUGR group, stages 1 and 2 were grouped together, indicating the resemblance of gut bacteria in the first 2 weeks of life among preterm infants without growth restriction. The gut microbiota composition shifted to stage 3 and then to stage 4, indicating gradual maturation. While in the EUGR group, although stages 1 and 2 were both grouped with their non-EUGR counterparts, stages 3 and 4 were both grouped with non-EUGR stage 3. The distances from EUGR stage 3/stage 4 to non-EUGR stage 3 were much smaller than those to non-EUGR stage 4 (Figure 1E). At the individual level, we could also observe a similar pattern between stage 3 and stage 4 in EUGR infants compared with the shifting pattern in non-EUGR infants (Supplementary Figure S3). These data indicated that the development of gut microbiota in EUGR infants was delayed compared with that in preterm infants without growth restriction.

### EUGR group related to lower obligate-to-facultative anaerobes ratio

**Figure 2:**
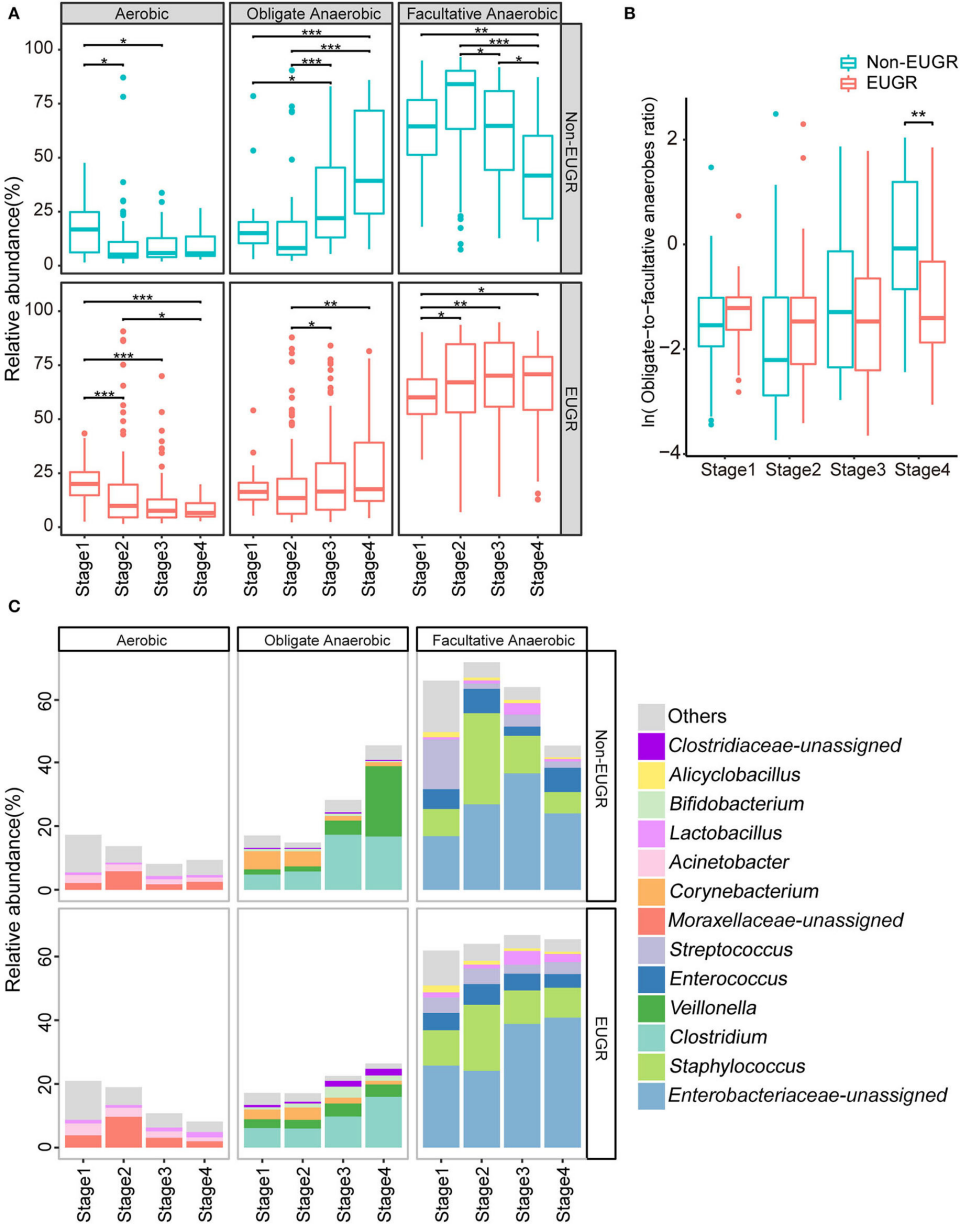
Dynamics of bacterial oxygen utilizing phenotypes in the EUGR and non-EUGR groups. **(A)** The dynamics of aerobic, obligate anaerobic, and facultative anaerobic in the four stages for the EUGR and non-EUGR groups. The differences among stages were tested using Kruskal–Wallis test, followed by Wilcoxon rank-sum test. (*p < 0.05; **p < 0.01; and ***p < 0.001). **(B)** The dynamics of obligate-to-facultative anaerobes ratio in the four stages for the EUGR and non-EUGR groups. The differences between the EUGR and non-EUGR groups were tested using Wilcoxon rank-sum test. **(C)** Microbial composition dynamics in the four stages at the genus level for preterm birth infants with and without EUGR.

As indicated from the stated results, we found that the dominant genera were mostly facultative bacteria, including *Enterobacteriaceae, Staphylococcus*, and *Enterococcus*. To explore the dynamics of bacterial phenotypes, we used BugBase to annotate the bacterial phenotypes, including aerobic, obligate anaerobic, and facultative anaerobic bacteria. Aerobes in both groups remained at a low level and tend to decline gradually. However, the tendencies of obligate anaerobes and facultative anaerobes in the EUGR and non-EUGR groups went in different directions. In the non-EUGR group, the obligate anaerobes gradually increased from stage 2 to stage 4 (*p* < 0.001) and positively correlated with chronological age (*r* = 0.43, *p* < 0.001), whereas the facultative anaerobes did just the opposite, decreasing from stage 2 to stage 4 (*p* < 0.001) and negatively correlated with chronological age (*r* = −0.26, *p* = 0.001) (Figure 2A, Supplementary Figure S4A). Compared with non-EUGR infants, the increase of obligate anaerobes is relatively small and the facultative anaerobes didn't change during stage 2 to stage 4 in EUGR infants, which suggests a delayed gut development. We clustered the bacterial oxygen phenotypes into one indicator by calculating the obligate-to-facultative anaerobes ratio, where a higher ratio indicates a larger proportion of obligate anaerobes with a smaller proportion of facultative anaerobes and vice versa. We observed that this ratio was significantly higher in the non-EUGR group in stage 4 (Figure 2B). To validate the relationship of obligate-to-facultative anaerobes ratio and growth restriction, we calculate this ratio by BugBase in a NICU cohort from Duke University (15). The result showed that the ratio was higher in the non-growth-failure group (Supplementary Figures S4B, C).

At the genus level, *Enterobacteriaceae-unassigned* and *Staphylococcus* were the dominant bacteria of facultative anaerobes in both groups. The relative abundance of *Staphylococcus* increased from stage 1 to stage 2 and then decreased at stages 3 and 4. The relative abundance of *Enterobacteriaceae-unassigned* increased from stage 1 to stage 3 in both groups. The divergence appeared at stage 4 as Enterobacteriaceae decreased in the non-EUGR group, whereas it increased in the EUGR group. In addition, the relative abundance of *Streptococcus* for all stages is stable in the EUGR group, whereas it decreased in the non-EUGR group. In contrast, *Clostridium* and *Veillonella* were the dominant bacteria of obligate anaerobes in both groups. The increase in the relative abundance of these two bacteria was slow in the EUGR group but rapid in the non-EUGR group (Figure 2C and Supplementary Figure S4D).

### Clinical characteristics associated with EUGR and bacteria oxygen phenotypes

By collecting data from medical records, we analyzed the dynamic trajectory of bacteria oxygen phenotypes using the Spearman's correlation test among these characteristics. The changes in bacterial oxygen phenotypes and delivery mode, antibiotics usage, and ventilator usage are shown in the Supplementary Figures S5A–C. We observed that the obligate-to-facultative anaerobes ratio significantly increased during hospitalization in infants exposed to antibiotics for <7 days rather than in those with over 1 week of antibiotic treatment (Supplementary Figure S5D). As for ventilator usage, we found that obligate-to-facultative anaerobes ratio significantly increased during hospitalization in infants without using ventilators (Supplementary Figure S5E). Regarding delivery mode, the correlation between the ratio and chronological age was significant in cesarean section, but not significant in spontaneous delivery (Supplementary Figure S5F), which is not consistent with the understanding that cesarean section was related to the unhealthy bacterial profile.

### The obligate-to-facultative anaerobes ratio associated with the long-term growth condition of preterm infants

**Figure 3:**
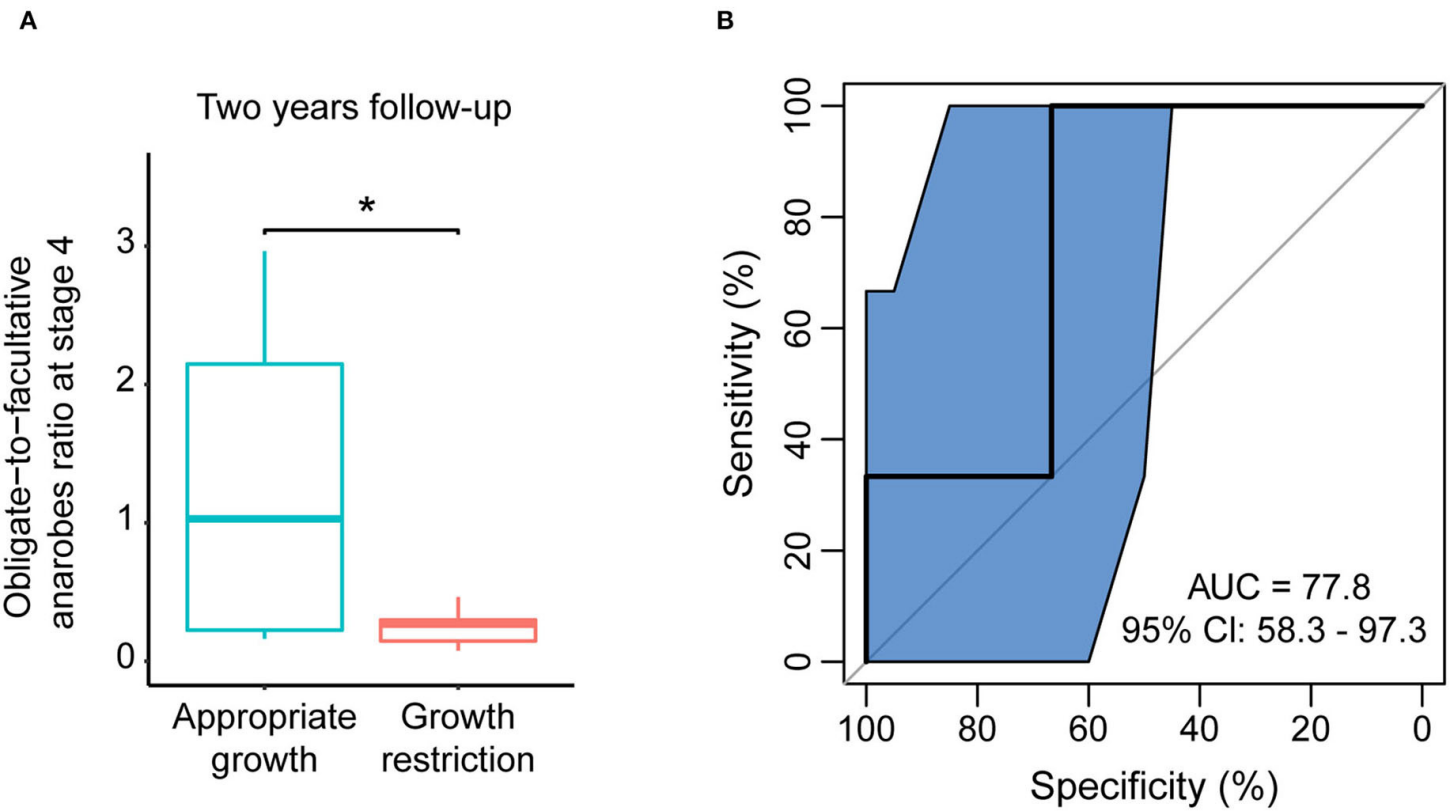
The obligate-to-facultative anaerobes ratio at stage 4 was associated with long-term growth restriction. **(A)** The obligate-to-facultative anaerobes ratio at stage 4 in the non-EUGR and EUGR groups at 2-year follow-up. *p < 0.05 by Wilcoxon rank-sum test. **(B)** Using the obligate-to-facultative anaerobes ratio at stage 4 as an indicator to distinguish long-term growth restriction in the EUGR group.

In the 2-year follow-up, we collected information regarding the weight of participants and calculated their growth condition according to the WHO Child Growth Standards' weight-for-age chart. Among the 42 that followed EUGR preterm infants, 10 remained in the growth restriction condition. In the 18 that followed non-EUGR preterm infants, one of the infants suffered growth restriction. To estimate the relationship between the obligate-to-facultative anaerobes ratio of stage 4 and long-term growth, we compared the obligate-to-facultative anaerobes ratio between infants with and without long-term growth restriction using Wilcoxon rank-sum tests and receiver operating characteristic curve analysis. In this analysis, we performed the analysis with 32 individuals who were both sampled at stage 4 and followed at 2-year follow-up. The ratio was significantly lower among the long-term growth restriction infants, and the area under the curve was 77.8% (Figures 3A,B). Then, we performed univariate and multivariate logistic regression analyses to access the odds ratios of bacteria oxygen phenotypes as a risk factor for the growth condition of 2-year follow-up. Although the statistic was at the edge of significance, we found that a higher obligate-to-facultative anaerobes ratio in the gut microbiota could be associated with a lower risk of long-term growth restriction in the univariate model and multivariate model adjusted by delivery mode, the duration of antibiotics, and ventilator use (Supplementary Table S3). These results indicated that the obligate-to-facultative anaerobes ratio could be associated with the long-term growth condition of preterm infants.

Considering that both chronological age and gestational age were associated with the maturity of the gut, we compared the gestational age between EUGR and non-EUGR groups, and no significance was detected. We further reanalyzed the data using corrected gestational age instead of chronological age and found that microbiota of the EUGR group were still clustered with the earlier stage of those in the non-EUGR group (Supplementary Figure S6A). We then performed Spearman's correlation between obligate-to-facultative anaerobes ratio and corrected gestational age in EUGR and non-EUGR groups, respectively, and found that the correlation coefficient in the non-EUGR group was higher than that in the EUGR group and the correlation in both groups showed statistical significance (Supplementary Figure S6B).

To estimate the relationship between obligate-to-facultative anaerobes ratio and long-term growth in corrected gestational age, we compared the mean obligate-to-facultative anaerobes ratio during 38–40 weeks for each individual between infants with and without long-term growth restriction using Wilcoxon rank-sum tests and receiver operating characteristic curve analysis. The ratio was not significantly lower among the long-term growth restriction infants, and the area under the curve was only 63.3%, indicating that obligate-to-facultative anaerobes ratio during stage 4 (chronological age 8–10 weeks) had a closer relationship with long-term growth than that during 38–40 weeks corrected gestational age (Supplementary Figures S6C, D).

## Discussion

In this study, we characterized the bacterial dynamic profiles of preterm infants and suggested that the development of gut microbiota in EUGR infants was stunted during hospitalization. Key support to this finding was that facultative anaerobes remained at a constantly high level, and obligate anaerobes remained at a low level in the gut microbiota of EUGR preterm infants compared with that of non-EUGR preterm infants. This phenomenon could be observed regardless of antibiotics and ventilator use. Although our data could not indicate the causal role of bacterial oxygen utilization in EUGR development, the follow-up results showed the potential of gut microbiota oxygen utilization ratio being an alerting signal for infant catch-up growth.

Recent studies have provided insights into the gut microbiota maturity during the early life and its association with healthy growth (8, 15, 21). However, the maturity is mainly assessed by estimated microbiota age using a machine learning algorithm, and the characteristics of stunted microbiota and their underlying explanation remain poorly understood. Li et al. found differences in the composition of intestinal flora between EUGR infants and very low birth weight infants by methods similar to ours. However, only meconium and feces on day 28 were collected with no follow-up in that study, which could not reflect the dynamic maturation process of gut microbiota (22). Generally, the high level of oxygen in the newborn gastrointestinal tract facilitates the colonization of facultative anaerobes, many of which are potential pathogens (23). As the early colonizers gradually consume oxygen in the gastrointestinal tract, obligate anaerobes consequently colonize in the new environment. In our data, the dynamics of the non-EUGR group meet the tendency of potential oxygen changes. However, in the EUGR group, a high proportion of facultative anaerobes and a low proportion of anaerobes remained during the whole hospitalization. Such a consistently high level of facultative anaerobes suggested a high oxygen level in the GI tract of EUGR preterm infants. Although the medical characteristics including ventilator and antibiotics usage could influence the oxygen concentrations or bacterial composition, the interactions between host and bacteria could contribute to growth restriction, including potential pathogens altering the host gut epithelial oxygenation and vulnerable immature infant GI tract suffering from pathogen translocation.

Firmicutes-to-bacteroidetes ratio (F/B ratio), a widely reported indicator in studies of obesity, has a close link with energy harvest and nutritional utilization, which recently has been reported to increase with age (24). However, such studies mainly involve the whole age group and few involve the dynamic changes of newborn infants from birth to 2 months of age. Furthermore, Proteobacteria, a phylum that accounted for a large proportion of the gut microbiota during the early life of infants and dramatically changed as the development of gut microbiota, would not be calculated in the F/B ratio. Besides, the performance of F/B ratio to predict long-term growth restriction was poor in our data (AUC: 61.1%). Therefore, compared with the F/B ratio, the obligate-to-facultative anaerobes ratio is more closely related to the dynamics of oxygen content during the infants' intestinal maturation and long-term growth.

In clinical practice, the postnatal course, nutritional management, and even medication were substantially differed by the gestational age of preterm birth infants. While a previous study suggested an association between gestational age and early life gut microbiota establishment, our study found that the delayed maturation of gut microbiota in EUGR infants was associated with both chronological age and corrected gestational age. Many studies have found the stunt of gut microbiota in preterm birth infants. Our data prompted that the profile of early life gut microbiota establishment is closely relative to the growth of preterm birth infants, which is consistent with a previous study (25). It is possible that the specific status or composition of gut microbiota could link to the onset of EUGR, which seems irrelevant to the clinical management and requires further studies to develop interventions for EUGR prevention.

Ventilatory oxygen support is necessary and is the most commonly used therapy for preterm infants to prevent neonatal respiratory distress syndrome and meet the needs of oxygen required for growth (26). However, less is known about how to balance the supply with need. The alteration of high epithelial oxygen consumption could lead to a decrease in beneficial obligate anaerobes and enrichment of facultative anaerobic bacteria, indicating a hallmark of gut dysbiosis (27). More days of ventilator use was associated with enriched Enterobacteriaceae (28). In this study, we observed the same trend in ventilator use of preterm infants. Antibiotics were applied to prevent or treat infections in preterm infants; however, early-life antibiotics use could result in many long-term adverse health effects. We didn't observe a significant increase in obligate-to-facultative anaerobes ratio across time in infants exposed to over 7 days of antibiotic treatment. This suggests that both ventilator use and antibiotics use could be associated with adverse effects for development, so was Khasawneh mentioned, while more research is required on rescues regarding the consequences of their necessary medical use (29). Studies with more detailed information (e.g., antibiotic types) are needed to explore the impact of clinical practice on the development of gut microbiota.

Although the criteria of EUGR by measuring anthropological indicators provide a convenient diagnosis for clinical practice, the lack of consensus on EUGR measurement still constrains research. Fenton et al. argued that the measurement and definition of growth charts could be arbitrary and unhelpful to medical practice (30); thus, new methods to monitor infants' growth are urgently needed. In this study, we suggest that the obligate-to-facultative anaerobes ratio was associated with the short-term and long-term growth of preterm infants. Whether this ratio could be a risk-alerting signal or even a target to guide probiotics therapies requires further research on a larger population.

### Limitations

Our study has some limitations. We only collected fecal samples from preterm infants without detecting the oxygen content in the infants' guts, which could provide direct evidence of the oxygen concentration and the relative consumption. However, despite the availability of the detection method, it would have been unethical to obtain these data by applying a cutting-edge oxygen sensor to NICU preterm infants. Another major limitation is that the sample size of our cohort was small, which limited us from examining the effect of innate factors and clinical practices in the development of preterm infants. The sample size also limited us from enabling a powerful analysis of antibiotic types and the relation between antibiotics and time of each sample. The oxygen utilization ratio was an observed risk factor for long-term growth on the boundary of significance, which suggests the requirement of future research on a larger population to examine this observation. It should be pointed out that our study is a monocentric study, and we used 16S rRNA gene sequencing for analysis, which limited our interpretation of species level and microbial function.

## Conclusion

Our findings indicate that the maturation of gut microbiota in the EUGR infants showed a significant delay compared with non-EUGR infants. This maturation delay is manifested in the consistently high level of facultative anaerobes and low level of obligate anaerobes. Furthermore, we suggest that the obligate-to-facultative anaerobes ratio during hospitalization may be correlated with long-term growth restriction.

## Data availability statement

The original contributions presented in the study are publicly available. This data can be found here: EBI: PRJEB45532.

## Ethics statement

The studies involving human participants were reviewed and approved by Medical Ethics Committee, Shenzhen Baoan Women's and Children's Hospital, Jinan University, Shenzhen, Guangdong Province, China. Written informed consent to participate in this study was provided by the participants' legal guardian/next of kin. Written informed consent was obtained from the individual(s), and minor(s)' legal guardian/next of kin, for the publication of any potentially identifiable images or data included in this article.

## Author contributions

EE-O, HZ, and PL designed the study, reviewed, and revised the manuscript. LM, YiZ, YS, YoZ, and ML collected the specimens. Y-EH, XS, and PL processed the samples. YH, XS, and PL searched the literature and drafted the manuscript. XS, SL, FH, JC, and PL analyzed the data. All authors approved the final version of the manuscript.

## Funding

This study was funded by the Research Foundation of Shenzhen Baoan Women's and Children's Hospital, Jinan University (BAFY 2021002).

## Conflict of interest

The authors declare that the research was conducted in the absence of any commercial or financial relationships that could be construed as a potential conflict of interest.

## Publisher's note

All claims expressed in this article are solely those of the authors and do not necessarily represent those of their affiliated organizations, or those of the publisher, the editors and the reviewers. Any product that may be evaluated in this article, or claim that may be made by its manufacturer, is not guaranteed or endorsed by the publisher.
