# Supplementary material for: Disrupted establishment of anaerobe and facultative anaerobe balance in preterm infants with extrauterine growth restriction
Source: Front Pediatr. 2022 Sep 6;10:935458. doi: 10.3389/fped.2022.935458 (PMC9486202; doi:10.3389/fped.2022.935458)
Supplement: Supplementary file 1 [file Data_Sheet_1.docx]

Supplementary Material

**Supplement Methods.** Detailed Methodology

**Table S1.** Demographics and clinical characteristics

**Table S2.** MaAsLin analysis determining EUGR associated genera in meconium after adjusting for delivery mode.

**Table S3.** The obligate-to-facultative anaerobes ratio associated with long-term growth outcome by logistic model

**Figure S1.** Dysbiosis of meconium gut microbiota in preterm birth patients with and without EUGR

**Figure S2.** Heatmap shows the relative abundance of predominant bacteria in each sample from individuals in EUGR and non-EUGR groups.

**Figure S3.** Dynamics of bacterial oxygen utilizing phenotypes.

**Figure S4.** Bacteria oxygen phenotypes in different subgroups.

**Figure S5.** Delay gut microbiome maturity in EUGR patients was also observed with corrected gestational age.

**Supplement Methods**

**Nutrition Strategies of Preterm Birth Infants**

For infants with birth weight below 1500g, umbilical vein catheter is administrated at the first week. After the first week, to avoid potential infection, the nutritional strategy turns to the peripherally inserted central catheter (PICC) to guarantee the Total Parenteral Nutrition (TPN) during the following hospital days. The volume of TPN was gradually decreased and ended finally when the preterm infants can be orally fed. Enteral Nutrition (EN) by the nasal feeding is a substitution for PICC in the first weeks. When the preterm infants can be orally fed, the nasogastric tube will be removed.

For infants with birth weight above 1500g but below 2500g, the nutritional strategy depends on the infant’s ability of swallowing. We will adjust the nutritional strategy between TPN and EN by the swallowing ability of infants.

Infants would receive the breast milk from their mother if available. And donated breast milk would be a substitution if the secretion of colostrum delays.

**Antibiotics Strategies of Mothers**

The mothers who were tested positive for Streptococcus agalactiae (GBS) or had premature rupture of membranes (PROM) for ≥18 hours would be given antibiotics before or during delivery.

**Antibiotics Strategies of Preterm Infants**

For the preterm infants, the antibiotics will be applied under two circumstances. First, if the infants are diagnosed sepsis, antibiotic will be applied until PCT drop to <0.05 ng/ml or the CRP < 10 mg/L, and the WBC and PLT turns to normal level. Second, when the pathogenic bacteria are detected in the blood, the antibiotic will be applied until the blood culture test is negative and double check negative within 24 hours.

**Ethical approval**

Written informed consents were obtained from all participants prior to this study. The protocol of the study was approved by medical ethics committee of the Shenzhen Baoan women’s and Children’s Hospital (LLSC-2021-1-13-KS).

**Calculation of** **Obligate-to-Facultative Anaerobes Ratio**

BugBase is a microbiome analysis tool that determines high-level phenotypes present in microbiome samples. We employed BugBase to determine the oxygen phenotypes in gut microbiome samples. Since Bugbase is based on Operational Taxonomic Unit (OTU) for 16S sequencing dataset, in our study, we assigned ASVs to GreenGenes database using BLAST, then applied the results to BugBase for corresponding oxygen phenotypes. For each sample, we would obtain the estimated level of aerobes, facultative anaerobes, and obligate anaerobes. The obligate-to-facultative anaerobes ratio was calculated using obligate anaerobes divided by facultative anaerobes.

**Clustering of time-dependent stage based on gut microbiota**

The mean relative abundance of predominant bacteria (relative abundance > 1%) for each time point of EUGR and non-EUGR groups were calculated. The R package *pheatmap* was used to demonstrate the change of bacterial abundance. The key stages of microbiome progression were determined by clustering the predominant bacteria of each time points in non-EUGR. The four chronological stages were stage 1 (0-4 days), stage 2 (1-2 weeks), stage 3 (3-7 weeks) and stage 4 (8-10 weeks). The samples of EUGR were also assigned to these four stages according to the sampling time, so as the samples from validation dataset.

**Table S1. Demographics and clinical characteristics**

| Variables | Total  (N=67) | Non-EUGR  (N=20) | EUGR  (N=47) | P-value |
| --- | --- | --- | --- | --- |
| Maternal age, years  (mean±SD) | 31.6±4.9 | 33.1±4.1 | 31.0±5.1 | 0.328 |
| Weeks of hospitalization stay  (mean±SD) | 8.0±3.6 | 8.1±3.7 | 7.8±3.3 | 0.717 |
| Gestational age |  |  |  | 0.271 |
| 24-28 weeks | 16(23.9%) | 6(30.0%) | 10(21.3%) |  |
| 28.1-32 weeks | 36(53.7%) | 12(60.0%) | 24(51.0%) |  |
| 32.1-36.6 weeks | 15(22.4%) | 2(10.0%) | 13(27.7%) |  |
| Birth weight |  |  |  | 0.131 |
| <1000g | 15(22.4%) | 2(10.0%) | 13(27.7%) |  |
| 1000-1499g | 29(43.3%) | 8(40.0%) | 21(44.7%) |  |
| 1500-2499g | 23(34.3%) | 10(50.0%) | 13(27.6%) |  |
| Delivery mode |  |  |  | 0.077 |
| V, Vaginal | 20(29.9%) | 9(45.0%) | 11(23.4%) |  |
| C, Caesarean | 47(70.1%) | 11(55.0%) | 36(76.6%) |  |
| Early antibiotic exposure (days) |  |  |  | 0.050 |
| 0 | 12(17.9%) | 3(15.0%) | 9(19.1%) |  |
| ≤7 | 29 (43.3%) | 13(65.0%) | 16(34.0%) |  |
| >7 | 26 (38.8%) | 4(20.0%) | 22(46.9%) |  |
| Ventilator use (days) |  |  |  | 0.031 |
| 0 | 32(47.8%) | 11(55.0%) | 21(44.7%) |  |
| ≤7 | 24(35.8%) | 3(15.0%) | 21(44.7%) |  |
| >7 | 11(16.4%) | 6(30.0%) | 5(10.6%) |  |
| Gender |  |  |  | 0.896 |
| Male | 41(61.2%) | 12(60.0%) | 29(61.7%) |  |
| Female | 26(38.8%) | 8(40.0%) | 18(38.3%) |  |
| Feeding intolerance (days) |  |  |  | 0.469 |
| 0 | 24(35.8%) | 6(30.0%) | 18(38.3%) |  |
| <14 | 26(38.8%) | 10(50.0%) | 16(34.0%) |  |
| ≥14 | 17(25.4%) | 4(20.0%) | 13(27.7%) |  |
| Type of feeding |  |  |  | 0.892 |
| <80% breast | 36(53.7%) | 11(55.0%) | 25(53.2%) |  |
| ≥80% breast | 31(46.3%) | 9(45.0%) | 22(46.8%) |  |
| Early-onset infection |  |  |  | 0.685 |
| No | 26(38.8%) | 9(45.0%) | 17(36.2%) |  |
| ^a^Early onset sepsis | 14(20.9%) | 3(15.0%) | 11(23.4%) |  |
| Pneumonia | 27(40.3%) | 8(40.0%) | 19(40.4%) |  |
| Maternal antibiotic exposure |  |  |  | 0.415 |
| Yes | 22(32.8%) | 8(40.0%) | 14(29.8%) |  |
| No | 45(67.2%) | 12(60.0%) | 33(70.2%) |  |
| Chorioamnionitis |  |  |  | 0.439 |
| Yes | 32(47.8%) | 11(55.0%) | 21(44.7%) |  |
| No | 35(52.2%) | 9(45.0%) | 26(55.3%) |  |
| Premature rupture of membranes |  |  |  | 0.167 |
| Yes | 22(32.8%) | 9(45.0%) | 13(27.7%) |  |
| No | 45(67.2%) | 11(55.0%) | 34(72.3%) |  |

^a^Early onset sepsis in this study was based on clinical diagnosis.

**Table S2. MaAsLin analysis** **determining EUGR associated genera in meconium after adjusting for delivery mode.**

| ^a^genus | coefficient | standard error | p values | q values |
| --- | --- | --- | --- | --- |
| Akkermansia | 3.043213775 | 0.833651302 | 0.000896554 | 0.050858736 |
| Knoellia | -2.307180631 | 0.645014707 | 0.001097992 | 0.050858736 |
| Variovorax | -1.664537667 | 0.46890471 | 0.001182761 | 0.050858736 |
| Rothia | -2.507499457 | 0.806499943 | 0.003849962 | 0.100089158 |
| Enterobacteriaceae-unassigned | 1.131889552 | 0.36660895 | 0.004073396 | 0.100089158 |
| Salmonella | -0.974552479 | 0.356615638 | 0.010012226 | 0.122299392 |
| Brachybacterium | -2.15223313 | 0.824621554 | 0.013509817 | 0.122299392 |
| Prevotella | 2.470916256 | 0.958383824 | 0.014582247 | 0.125407324 |
| Moraxellaceae-unassigned | 1.346566807 | 0.531798418 | 0.016279805 | 0.133339356 |
| Enterococcus | 1.428327414 | 0.587287791 | 0.0206026 | 0.138309124 |
| Lactobacillus | 0.967327322 | 0.402583164 | 0.022052721 | 0.138309124 |

^a^Only genera with significance (q values < 0.2) were showed in this table.

**Table S3. ORs, RRs, and 95% CIs of long-term growth restriction according to the obligate-to-facultative anaerobes ratio at the stage 4**

| The obligate-to-facultative anaerobes ratio | ^a^Lower (n = 15) | ^a^Higher (n = 17) | | |
| --- | --- | --- | --- | --- |
| Mean and range | 0.207 (0.078-0.300) | 2.223 (0.464-6.361) | | |
| ^b^Case: n (%) | 5 (33.3%) | 1 (5.88%) | | |
| **Models** |  | OR (95% CI) | ^h^Wald's test | ^h^LR-test |
| ^c^Model 1 | Reference | 0.1 (0.01,1.03) | *p* = 0.053 | ***p* = 0.026** |
| ^d^Model 2 | Reference | 0.09 (0.01,1.07) | *p* = 0.057 | ***p* = 0.03** |
| ^e^Model 3 | Reference | 0.1 (0.01,1.06) | *p* = 0.056 | ***p* = 0.029** |
| ^f^Model 4 | Reference | 0.1 (0.01,1.04) | *p* = 0.053 | ***p* = 0.026** |
| ^g^Model 5 | Reference | 0.09 (0.01,1.08) | *p* = 0.058 | ***p* = 0.031** |

^a^Higher and lower obligate-to-facultative anaerobes ratio was defined by using 0.35 as cutoff to divided 32 infants into two groups with comparable sample size (17 vs 15).

^b^The number and percentage of cases that suffered long-term growth restriction.

^c^Model 1: univariate logistic regression model.

^d^Model 2: multivariate logistic regression model that adjusted for delivery mode (vaginal delivery/cesarean delivery).

^e^Model 3: multivariate logistic regression model that adjusted for duration of antibiotics use (quantiles).

^f^Model 4: multivariate logistic regression model that adjusted for duration of ventilator use (quantiles).

^g^Model 5: multivariate logistic regression model that adjusted for duration of antibiotics use (quantiles), duration of ventilator use (quantiles) and delivery mode (vaginal delivery/cesarean delivery).

^h^The significance was tested by Wald's test and the likelihood ratio test (LR-test)


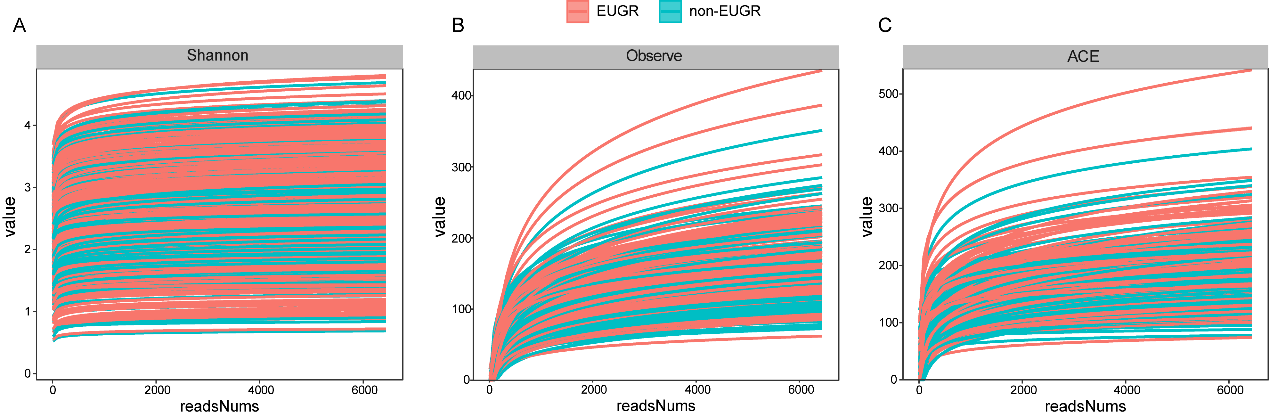


**Figure S1. Effect of rarefaction curves in alpha diversity.** 16s ribosomal RNA gene sequencing showed changes in the rarefaction curve, including Shannon Index (A), Observed Amplicon Sequence Variants (ASVs) (B), and ACE Index (C).


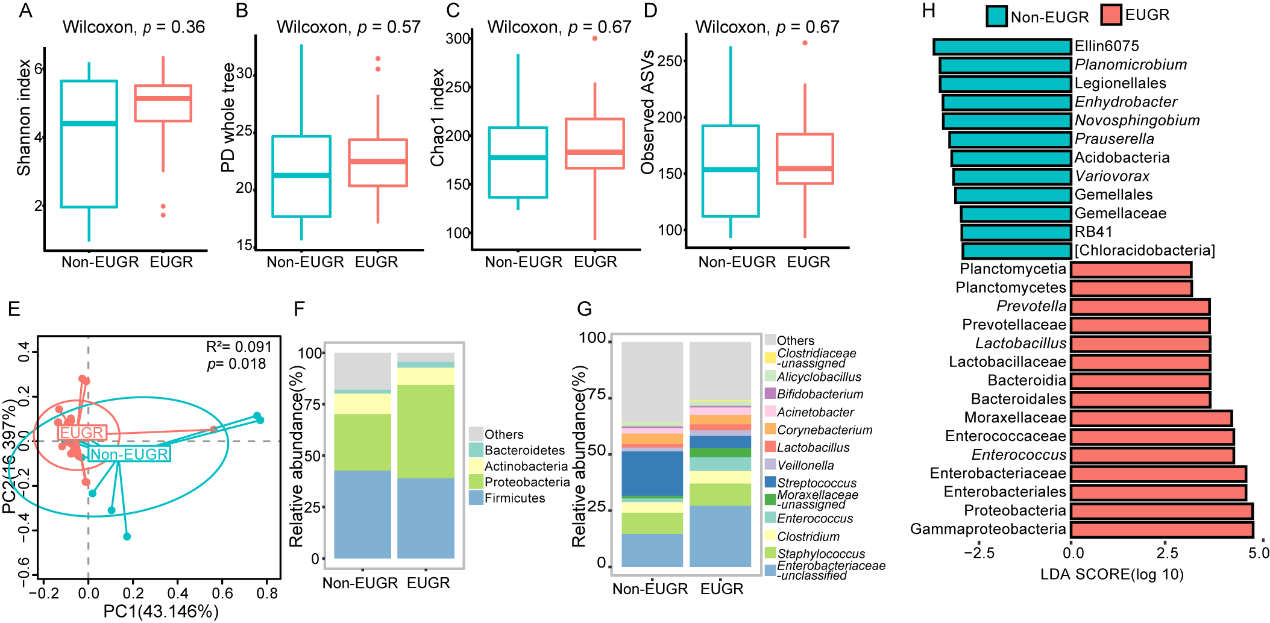


**Figure S2. Dysbiosis of meconium gut microbiota in preterm birth patients with and without EUGR.** (A-D) Comparison of alpha-diversity indices (Shannon Index, PD-whole-tree Index, Chao1 Index and Observed Amplicon Sequence Variants (ASVs) between preterm birth patients with and without EUGR. Wilcoxon rank-sum test. (E) Principal coordinate analysis based on unweighted UniFrac distances between preterm birth patients with and without EUGR by PERMANOVA. (F, G) Microbial composition at the phylum level and genus level of preterm birth patients with and without EUGR. (H) Linear discriminant analysis effect size (LEfSe) analysis identified different taxa between preterm birth patients with and without EUGR. The LDA scores (log10) > 3.0 are listed.


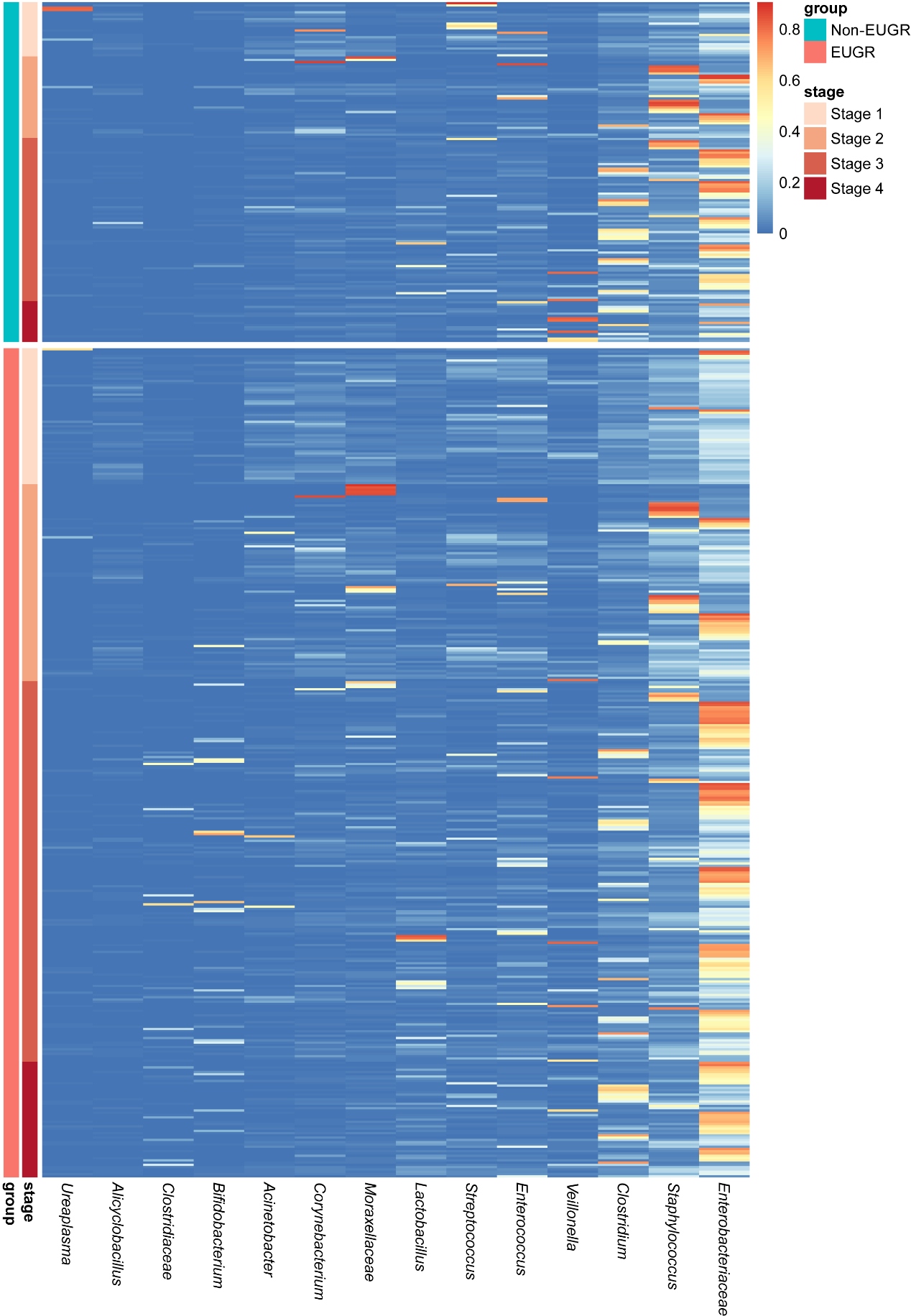


**Figure S3. Heatmap shows the relative abundance of predominant bacteria in each sample from individuals in EUGR and non-EUGR groups.**


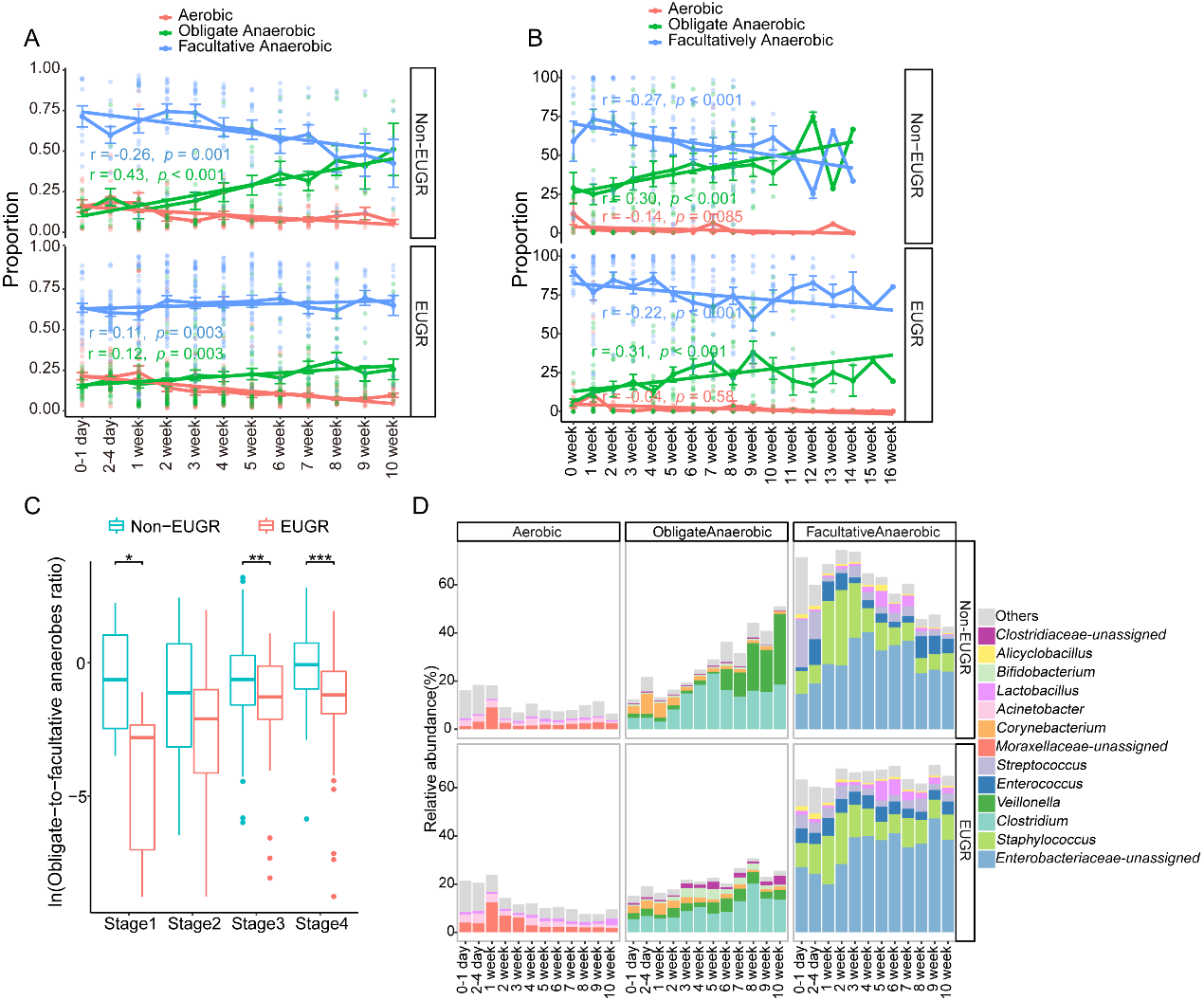


**Figure S4.** **Dynamics of bacterial oxygen utilizing phenotypes.** (A) the relative abundance of aerobe, obligate anaerobe and facultative anaerobe during the hospitalization in preterm infants with and without EUGR. (B) Validation data from Duke University about the relative abundance of aerobe, obligate anaerobe and facultative anaerobe in a NICU cohort. (C) Validation data from Duke University about the obligate-to-facultative anaerobes ratio in a NICU cohort. (D) Microbial composition of each bacterial oxygen utilizing phenotypes at the genus level in preterm infants with and without EUGR.


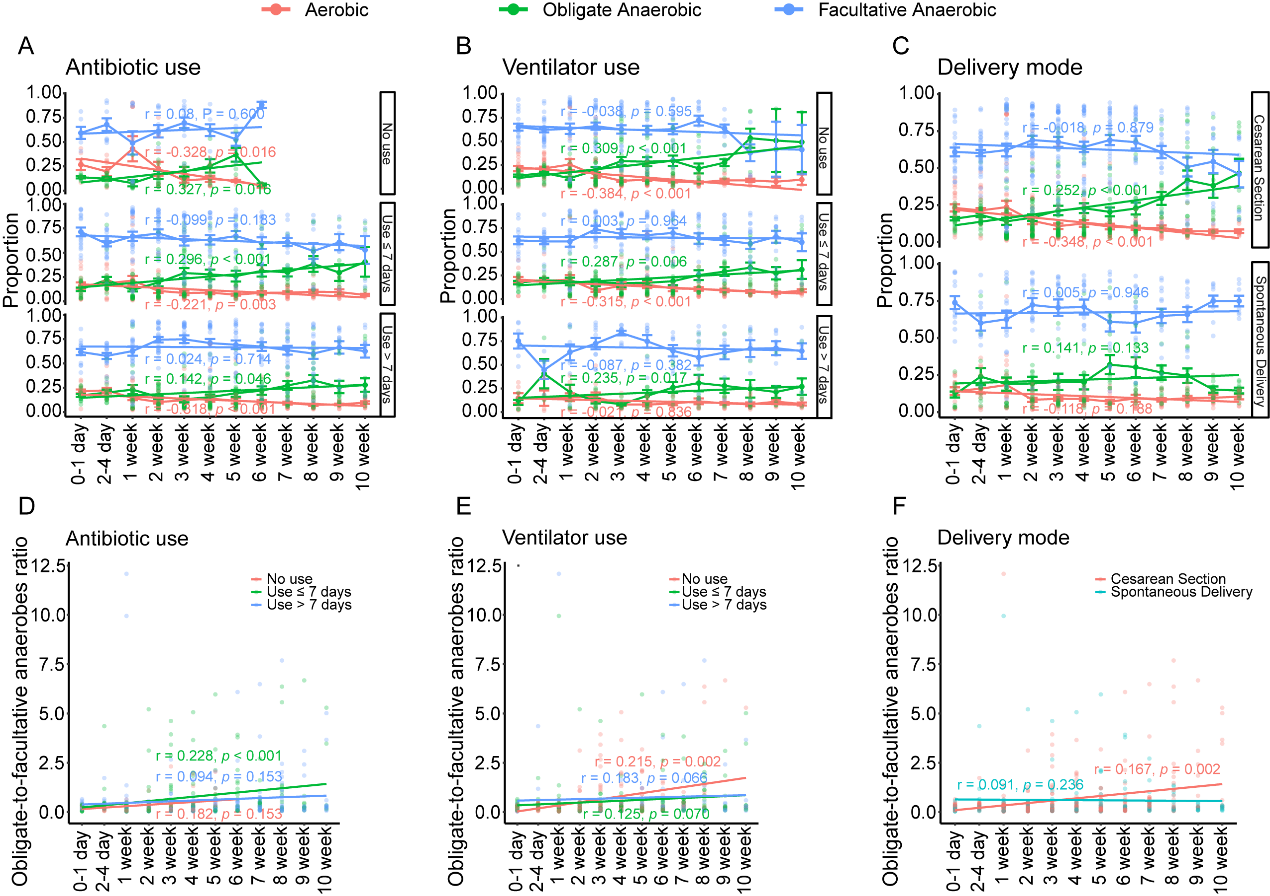


**Figure S5. Bacteria oxygen phenotypes in different subgroups.** (A-C) The relative abundance of aerobe, obligate anaerobe and facultative anaerobe during the hospitalization in subgroups of antibiotics usage, ventilator usage and delivery mode. (D-F) Obligate-to-facultative anaerobes ratio during the hospitalization in subgroups of antibiotics usage, ventilator usage and delivery mode. The correlations were tested by Spearman’s correlation test.
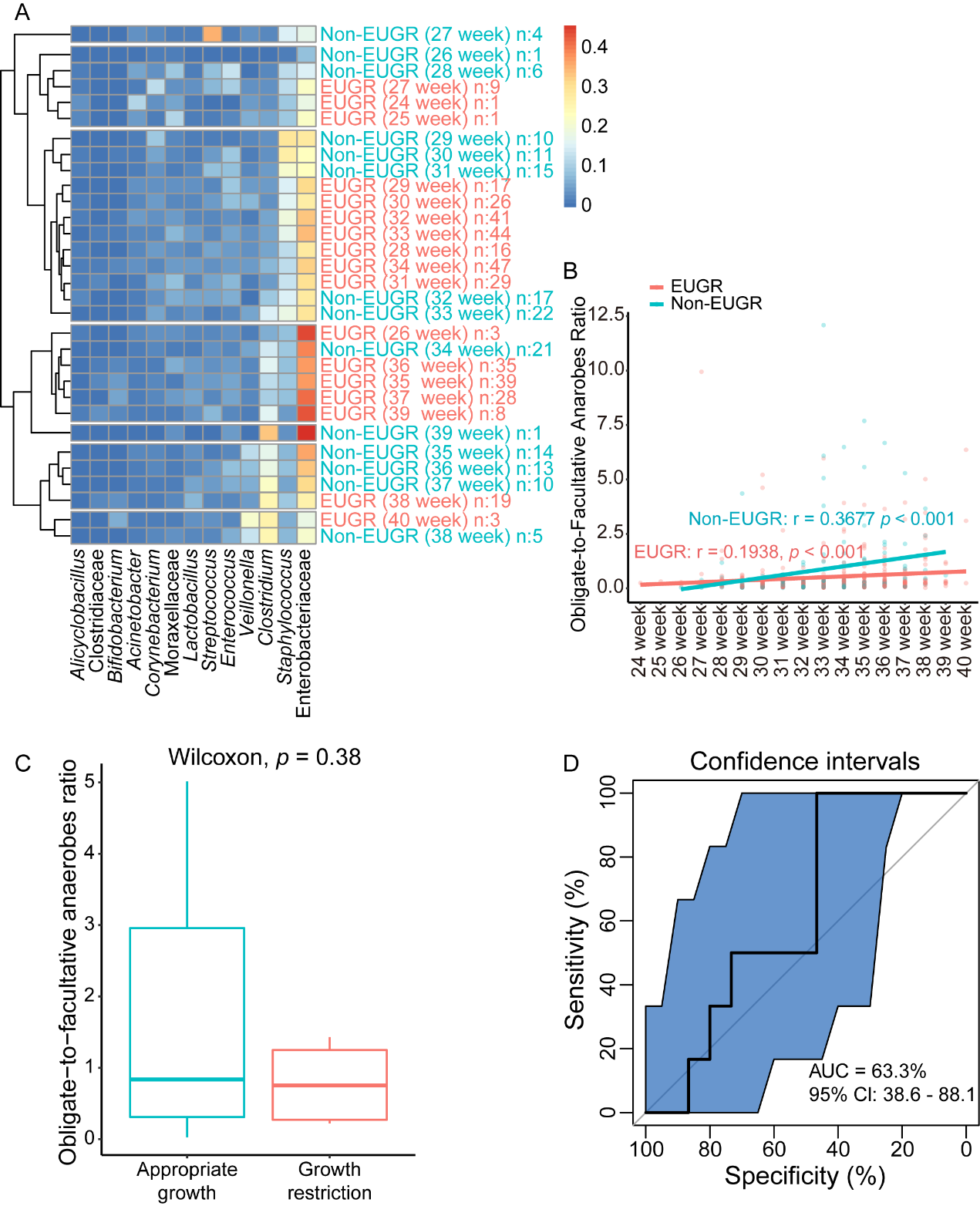


**Figure S6. Delay Gut Microbiome Maturity in EUGR Patients was also Observed with corrected Gestational Age.** (A) Heatmap shows the predominant bacteria in the EUGR group clustered together with the non-EUGR group by corrected gestational age. (B) Correlation analysis shows the obligate-to-facultative anaerobes ratio time-varying of EUGR groups and non-EUGR groups by corrected gestational age. The correlations were tested by Spearman’s correlation test. (C) The obligate-to-facultative anaerobes ratio at the corrected gestational age of 38-40 weeks in the appropriate growth groups and growth restriction groups. The difference between groups was tested by Wilcoxon rank-sum test. (D) Using the obligate-to-facultative anaerobes ratio at the corrected gestational age of 38-40 weeks as an indicator to distinguish long-term growth restriction in the EUGR group.
